# Tennis Elbow Diagnosis Using Equivalent Uniform Voltage to Fit the Logistic and the Probit Diseased Probability Models

**DOI:** 10.1155/2015/585180

**Published:** 2015-08-25

**Authors:** Tsair-Fwu Lee, Wei-Chun Lin, Hung-Yu Wang, Shu-Yuan Lin, Li-Fu Wu, Shih-Sian Guo, Hsiang-Jui Huang, Hui-Min Ting, Pei-Ju Chao

**Affiliations:** ^1^Medical Physics and Informatics Laboratory of Electronics Engineering, National Kaohsiung University of Applied Sciences, 415 Chien Kung Road, San-Min District, Kaohsiung 80778, Taiwan; ^2^Institute of Photonics and Communications, National Kaohsiung University of Applied Sciences, Kaohsiung 80778, Taiwan; ^3^Department of Orthopedic, Kaohsiung Municipal Min-Sheng Hospital, Kaohsiung 80276, Taiwan; ^4^Department of Electronics Engineering, National Kaohsiung University of Applied Sciences, Kaohsiung 80778, Taiwan; ^5^Department of Plastic Surgery, Kaohsiung Municipal Min-Sheng Hospital, Kaohsiung 80276, Taiwan; ^6^Department of Radiation Oncology, Kaohsiung Chang Gung Memorial Hospital and Chang Gung University, College of Medicine, Kaohsiung 83342, Taiwan

## Abstract

To develop the logistic and the probit models to analyse electromyographic (EMG) equivalent uniform voltage- (EUV-) response for the tenderness of tennis elbow. In total, 78 hands from 39 subjects were enrolled. In this study, surface EMG (sEMG) signal is obtained by an innovative device with electrodes over forearm region. The analytical endpoint was defined as Visual Analog Score (VAS) 3+ tenderness of tennis elbow. The logistic and the probit diseased probability (DP) models were established for the VAS score and EMG absolute voltage-time histograms (AVTH). TV_50_ is the threshold equivalent uniform voltage predicting a 50% risk of disease. Twenty-one out of 78 samples (27%) developed VAS 3+ tenderness of tennis elbow reported by the subject and confirmed by the physician. The fitted DP parameters were TV_50_ = 153.0 mV (CI: 136.3–169.7 mV), *γ*_50_ = 0.84 (CI: 0.78–0.90) and TV_50_ = 155.6 mV (CI: 138.9–172.4 mV), *m* = 0.54 (CI: 0.49–0.59) for logistic and probit models, respectively. When the EUV ≥ 153 mV, the DP of the patient is greater than 50% and vice versa. The logistic and the probit models are valuable tools to predict the DP of VAS 3+ tenderness of tennis elbow.

## 1. Introduction 

Lateral epicondylitis, also named as tennis elbow, is a condition where the outer part of the elbow becomes sore and tender. It is a very common clinical occurrence, with many working population being involved [1]. While the common name “tennis elbow” suggests a strong link to racquet sports [2], this condition can also be caused by other sports such as swimming and climbing, the work of manual workers and waiters, as well as activities of daily living [3–5]. Tennis elbow is an overuse injury occurring in the lateral side of the elbow region, but more specifically it occurs at the common extensor tendon that originates from the lateral epicondyle. The acute pain that a person might feel occurs as one fully extends the arm.

While the rest is the most frequently prescribed treatment for most soft tissue injury, for athletes and industrial workers, it may not be possible to cease or severely limit the activity that caused the condition to appear. Nirschl has proposed using the term tendonosis due to the lack of inflammation accompanying common lateral tennis elbow [6]. With an injury such as lateral epicondyle tendonitis, because the pain associated with the injury may actually encourage counterproductive muscle activation [7]. It is therefore important to determine if the patient is really belonging to this diagnosis, and how to define the possible detrimental muscle activation patterns accompanying this disease. In Nirschl's study, they had demonstrated the surface electromyography (EMG) signal of tennis elbow with difference in muscular activation time, frequency, duration, and earlier onset time [7].

When diagnosing the tennis elbow, patient-reported of pain is usually sufficient for a clinician to make a recommendation of treatment [8]. How to determine if muscle usage patterns have been altered by the injury is an important issue. EMG data from the extensor carpi radialis brevis (ECRB) showing increased activity during specific wrist movements would indicate that muscular strain on the damaged soft tissue, usually the hyaline region of the ECRB [9]. This evidence indicates that in tennis elbow patients the muscle electric activity is different from the normal groups. However, other muscle groups may also be affected. Generally, tennis elbow affects mostly wrist extensor muscles. These muscles are innervated by the radial nerve, and therefore differential diagnosis would include radial neuropathy, cervical radiculopathy (C6) and muscle or tendon pathology. It is well known that different neural muscular condition can be characterized by different EMG patterns. However, to our best knowledge, there is no consistent feature of EMG for diagnosis of tennis elbow [10]. In previous studies, changes in motor unit morphology or firing pattern associated with underlying pathophysiology are described. Shape characteristics of motor unit potentials (MUPs) provide insight into the underlying pathophysiology of neuromuscular diseases [11]. In myopathies, classic EMG findings are MUPs with reduced durations and amplitudes due to loss of muscle fibers or fibrosis [12]. There is also increased complexity in the MUP waveforms, which may be associated with atrophic or regenerating muscle fibers or with temporal dispersion among muscle fiber potentials due to fiber diameter variations [13]. In neuropathies, classic EMG findings include increases in MUP duration and amplitude caused by increased fiber number and density as orphaned muscle fibers receive axonal sprouts from healthy axons. In this case, the number of turns and phases may either be normal or increased [14].

Since the pathogenesis of this condition is still unknown, diagnosis and treatment of this disease are often complicated by reliance on self-reported of the patient himself. Recently, the clinician's ability to more accurately view the damage through quantitative means, such as MRIs, has been greatly improved. However, such evaluation is expensive and not cost-effective. Recently many studies have focused on predictive modelling for diseased probability (DP) on various anatomic sites and reporting known models parameters [15, 16]. To our knowledge, there is no study describing DP of EMG signal measured from the epicondyle for tenderness of tennis elbow endpoint. In this study, we introduce two widely used DP models, namely, the logistic and the probit, to quantify the relationship between tenderness of tennis elbow and EMG intensity measured from the epicondyle, which potentially identify a more specific EMG relationship.

So, we aim to determine the best-fit parameters of these well-known and established DP models and introduce the model best describing the EMG intensity-response relationship of the epicondyle for tennis elbow endpoint. Therefore to determine if simple surface EMG analysis of muscles associated with lateral tennis elbow could be used to assist in accurately diagnosing this common soft tissue injury.

## 2. Materials and Methods

### 2.1. Participants

**Figure 1:**
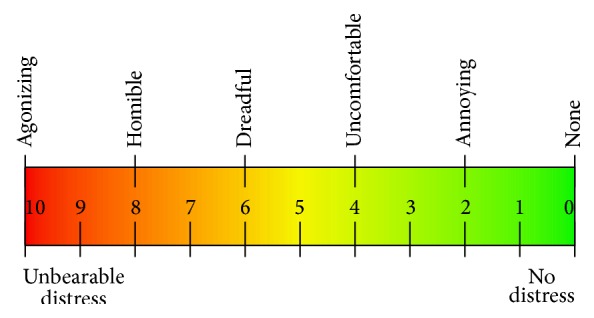
Visual Analog Score (VAS) for assessing the severity of tenderness.

In this study, a total of 39 subjects were enrolled. Their demographic data including sex, age, and disease status are recorded. For each diseased subject, the involved side was used as desired sample and the other side was used as normal control. Patients with bilateral elbow involved were excluded from analysis. Every tennis elbow patient will have elbow X-ray for anterior-posterior and lateral views to rule out severe degenerative disease. For every patient, the detailed history was taken by the physician. Physical examination including palpation for tender point was also performed by the physician. The severity of tenderness is assessed by Visual Analog Score (VAS) as shown in Figure 1. VAS zero score is the least pain, while the VAS 10.0 points is the worst pain. During VAS score acquisition, there is no verbal or conscious guidance provided by the nurses or physicians. The VAS score was directed by patient self-reported. The analytical endpoint was defined as VAS 3+ tenderness of tennis elbow. This study was approved by the institutional review boards of the Min-Sheng hospital (KMMH-IRB-10201).

### 2.2. Electrodes Placement

When acquiring the surface EMG (sEMG) signal, patients were put in sitting position. The electrodes of EMG machine was attached to the standard EKG led pads then applied to skin surface. The surface electrode pad was composed of AgCl. The three electrodes of EMG machine were put on:

1. the muscle belly of ECRB,
2. distal radius Lister's tubercle of dorsal wrist,
3. ulnar styloid over lateral side of wrist.

**Figure 2:**
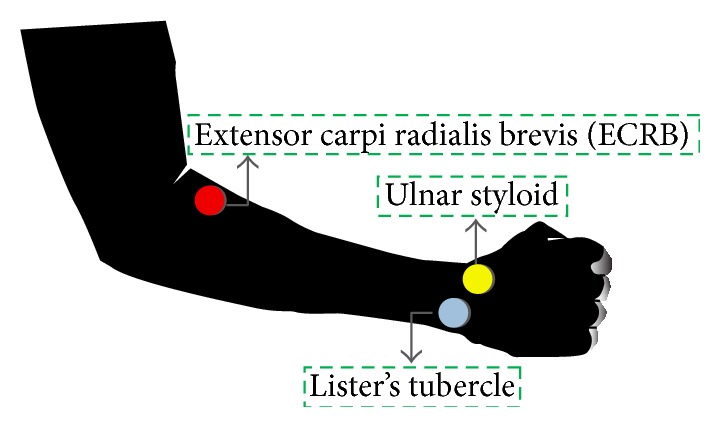
The two electrodes position over wrist: the Lister's tubercle is marked as blue circle and ulnar styloid is marked as yellow circle. The other electrode is put over ECRB belly site as red circle.

The electrode over ulnar styloid was set as the reference. The electrodes' position and relevant human anatomy was shown in Figure 2.

### 2.3. Experimental Protocol

**Figure 3:**
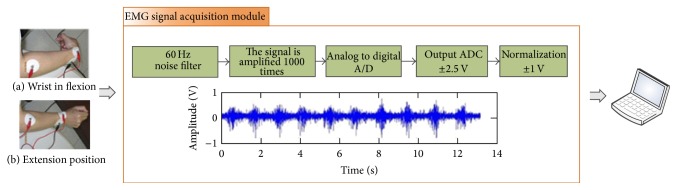
Setup of our devices for surface EMG signal acquisition when (a) wrist in flexion and (b) extension position, EMG signal acquisition module (EMG-01, Raising Technology Co., Ltd., Kaohsiung, Taiwan).

Subject in test was told to hold fist. Then active wrist flexion extension was asked to perform by patient himself. One cycle of range-of-motion (ROM) is defined as one flexion and one extension. Wrist position in flexion and extension is shown in left part of Figure 3. The duration of one cycle was kept to around 1.3 seconds. Total ten cycles around 13 s was recorded. The other side upper limb was repeated as the same protocol.

The sEMG signal was amplified and filtered by an EMG signal acquisition module (EMG-01, Raising Technology Ltd., Kaohsiung, Taiwan). Output end of the amplifier was normalized to ±1 voltage then connected to the audio line-in port of personal computer. The setup of our device is shown in Figure 3. The data acquisition process is performed with Matlab software. The signal is input as standard vector form after taking its absolute value in Matlab programming language.

**Figure 4:**
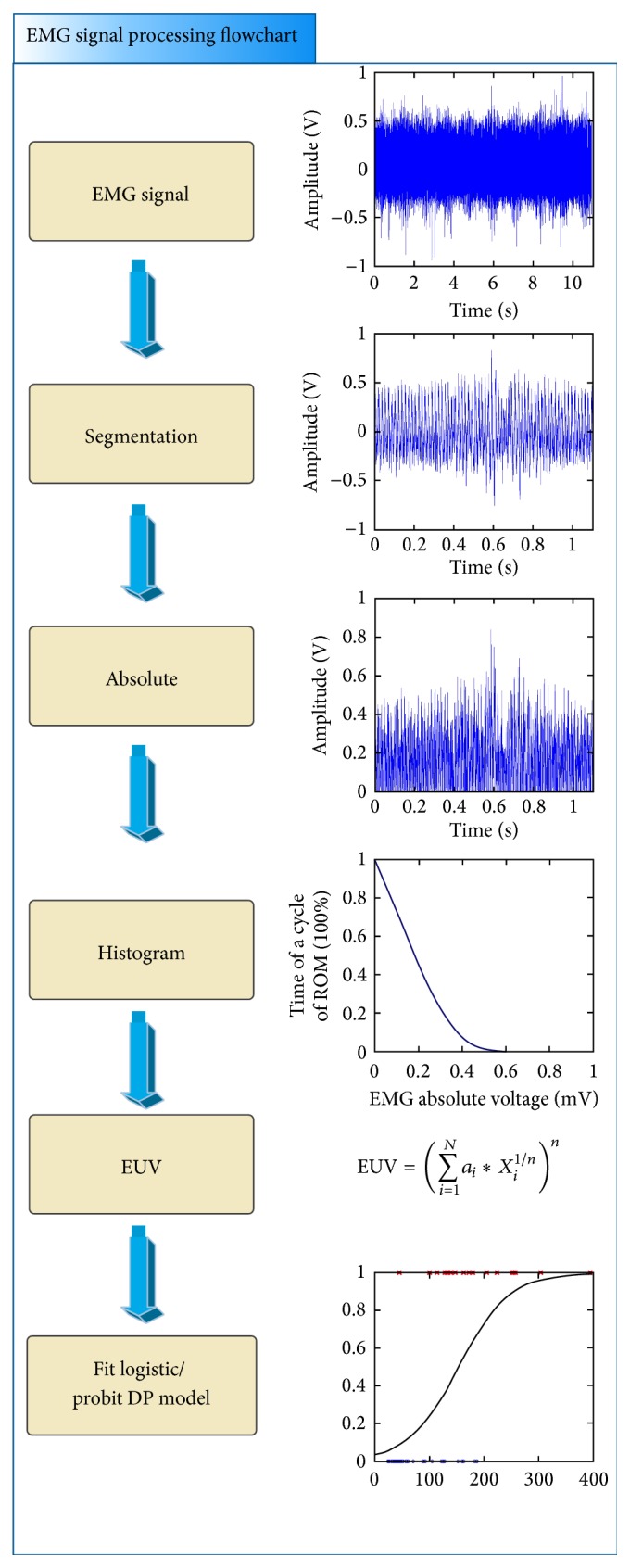
EMG signal processing flowchart. EMG: electromyographic; EUV: equivalent uniform voltage; DP: diseased probability.

In order to provide robust algorithm for treating the sEMG signal, a nonparametric histogram method is used [17, 18]. In this study, we call it EMG absolute voltage-time histograms (AVTH). The total period for an EMG signal is normalized. The concept of AVTH is similar to the concept of dose-volume histogram (DVH) [17]; it is used widely in radiation treatment planning. The horizontal axis of AVTH plot is the voltage intensity of signal; it presented the absolute value of the normalized EMG signal (0-1V), while its vertical axis represents the cumulative histogram in complement, that is, one minus cumulative histogram [18]. An EMG signal processing flowchart is illustrated in Figure 4.

AVTH are used to plot the density of data which comes from the EMG absolute voltage, it is a graphical representation of the distribution of data. And it is a representation of tabulated frequencies, shown as adjacent rectangles, erected over discrete intervals (bins), with an area proportional to the frequency of the observations in the interval. The bin size used in this study is 1 mV. The height of a rectangle is also equal to the frequency density of the interval, that is, the frequency divided by the width of the interval. The total area of the histogram is equal to the number of data. AVTH is normalized displaying relative frequencies. If the total area of a histogram used for probability density is normalized to 1.

Another nonparametric method is calculating the equivalent uniform voltage (EUV) for the corresponding AVTH. The definition of EUV is as (1) below:

(1) $$\begin{array}{c} \text{EUV}={\left(\overset{N}{\underset{i=1}{\sum }}a_{i}*X_{i}^{1/n}\right)}^{n}, \end{array}$$

where *N* is the length of vector,* n* is a parameter that describes the magnitude of the period effect, and* a*_*i*_ is the period of the voltage bin that corresponds to voltage* X*_*i*_ in the differential AVTH. Therefore the EUV data are used to fit the logistic and the probit predictive models for the diseased risk of tennis elbow.

#### 2.3.1. Logistic Model

A logistic model was used to fit EUV-response for the DP of VAS 3+ tenderness of tennis elbow as a function of mean EMG EUV measured from the epicondyle according to the following formula:

(2) $$\begin{array}{c} {\text{DP}}_{\text{logistic}}=\frac{\exp\left(4\gamma_{50}\left(\text{EUV}/{\text{TV}}_{50}-1\right)\right)}{1+\exp\left(4\gamma_{50}\left(\text{EUV}/{\text{TV}}_{50}-1\right)\right)}, \end{array}$$

where EUV is mean intensity measured from the epicondyle; TV_50_ is the threshold EUV predicting a 50% risk of disease; and *γ*_50_ is normalized slope of the EUV-response curve, that is, change in DP_logistic_ in units of percent per 1% change in EUV [19, 20]. The best-fitting values for TV_50_ and *γ*_50_ were found using maximum likelihood analysis, and the 95% confidence intervals were found using the profile likelihood method [16, 21]. The software used for the fitting process was Matlab (R2009; MathWorks, Natick, MA).

#### 2.3.2. Probit Model

The family of probit models is the most widely used phenomenological approach. The probit model was described by three parameters:* n*,* m*, and TV_50_, and the* n* was set to 1 in this study. According to this model, DP_probit_ is described as the following equations:

(3) $$\begin{array}{c} {\text{DP}}_{\text{probit}}=\frac{1}{\sqrt{2\pi }}\overset{t}{\underset{-\infty }{\int }}\exp\left(-x^{2}/2\right)dx,\\ t=\frac{\text{EUV}-\;{\text{TV}}_{50}}{m\cdot {\text{TV}}_{50}}, \end{array}$$

where the parameter *m* is a unitless model parameter for describing the slope of the EUV-response curve. The definition of EUV and TV_50_ is the same as the above. The best-fitting values for TV_50_ and *m* were determined by using maximum likelihood estimation, and 95% confidence intervals were found using the profile likelihood method with exact binomial confidence intervals for each bin. A criterion TV_25_ is suggested for the threshold EUV producing a 25% diseased probability within a specific period of time. Such criterion may be a useful index for early disease detection.

#### 2.3.3. Performance Evaluation

The system performance can be checked by using the area under the receiver operating characteristic curve (AUC), scaled Brier score, Nagelkerke* R* ^2^, and Hosmer-Lemeshow test, chi-square goodness-of-fit test. We also report negative predictive value (NPV) [16], which gives the rate of prevention of the DP of VAS 3+ tenderness of tennis elbow. The equation used for NPV is

(4) $$\begin{array}{c} \text{NPV}=\frac{S\times \left(1-P\right)}{\left(1-S\right)\times P+S\times \left(1-P\right)}, \end{array}$$

where* S* means specificity and* P* presents prevalence. A high NPV would support the validity of a suggested criterion (TV_25_). This analysis was performed for both TV_50_ and TV_25_ criteria. Statistical analyses were performed using SPSS 19.0.

## 3. Results 

In total, 78 hands from 39 subjects are enrolled in this study. Demographic data of our test subjects is shown in Table 1. Figure 2 shows the electrodes placement with corresponding anatomic positions. The equipment for acquiring the sEMG signal is shown in Figure 3. This demonstrated the wrist in (a) full flexion and (b) full extension position.

**Figure 5:**
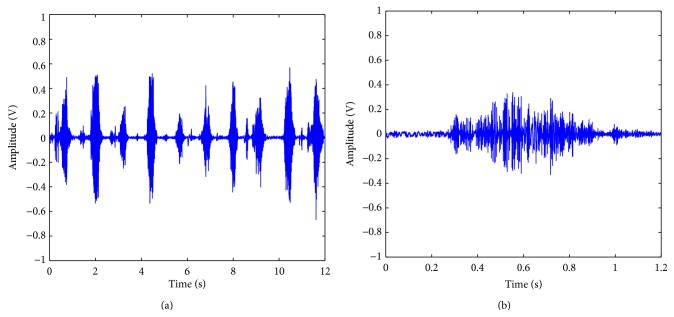
(a) Surface EMG signal from normal control subject with ten cycles of wrist ROM. (b) Detailed view of one surface EMG cycle of wrist ROM.

**Figure 6:**
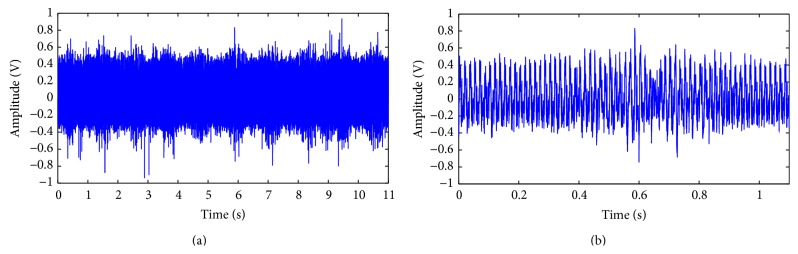
(a) Surface EMG signal from diseased patient with ten cycles of wrist ROM. (b) Detailed view of one surface EMG cycle of wrist ROM.

**Figure 7:**
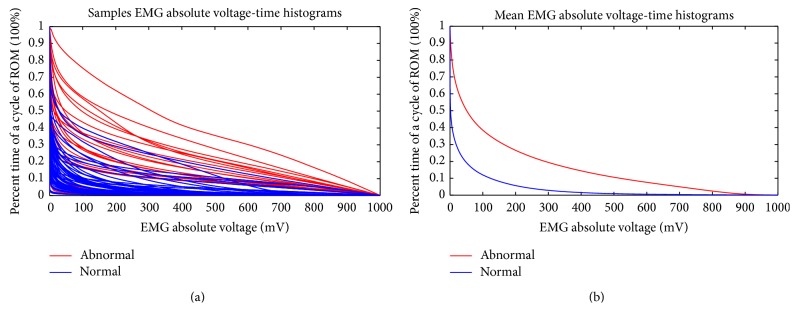
(a) The all-samples EMG absolute voltage-time histograms (VTH) signals. (b) The mean samples EMG absolute VTH signals for group with/without VAS 3+ tenderness of tennis elbow. Blue line is of normal subjects. Red lines represent diseased subjects.

One normal control signal with ten cycles of wrist ROM is shown in Figure 5(a).  Figure 5(b) is the detailed view of one cycle. One sample of diseased signal is shown in Figure 6(a), and Figure 6(b) is the detailed view.  Figure 7 shows all samples EMG AVTH signals and (b) shows the mean samples EMG AVTH signals for group with/without VAS 3+ tenderness of tennis elbow. Blue line is of normal subjects and red lines represent diseased subjects.

**Figure 8:**
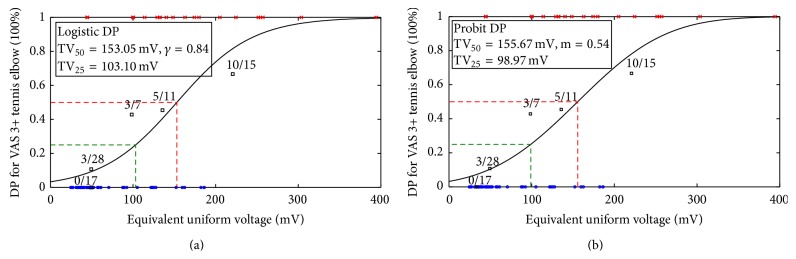
The diseased probability (DP) models developed (a) logistic and (b) probit model.

The fitted DP curves (logistic and probit models) for the VAS 3+ tenderness of tennis elbow are shown in Figures 8(a) and 8(b). DP fitted parameters were TV_50_ = 153.0 mV (CI: 136.3–169.7 mV),  *γ*_50_ = 0.84 (CI: 0.78–0.90) and TV_50_ = 155.6 mV (CI: 138.9–172.4 mV),* m *= 0.54 (CI: 0.49–0.59) for Logistic and probit models, respectively (Table 2). This result illustrates when the EUV ≥ 153 mV, the diseased probability of the patient is greater than 50%. A suggested guideline TV_25_ for the threshold EUV producing a 25% complication rate within a specific period of time was TV_25_ = 103.1 mV (Logistic) and TV_25_ = 98.9 mV (probit), respectively.

The overall performance for the DP models for the VAS 3+ tenderness of tennis elbow in terms of the AUC, Hosmer-Lemeshow test, Brier score, and* R* ^2^ was satisfactory with the expected values (Table 3). The AUC for the DP models were 0.84 (95% CI: 0.74–0.94) for both logistic and probit models. For the chi-square goodness-of-fit test, quantitatively, this is manifest by the small values of *χ* ^2^ for both models and indicates lack of statistical difference between predictive risk and the observed outcome. NPVs are given in Table 3. The accuracy rates are 0.78 for both logistic and probit models.

## 4. Discussions

Elbow lateral epicondylitis is the most common tendinosis disease and it affects a lot of people, especially the elderly and heavy workers [5, 6, 8, 22]. Nowadays, physical examination and history taking remain the gold standard for diagnosis of tennis elbow. The physical examination elucidates how tender point is located over the extensor muscle origin. At the same time, the severity of tenderness is also recorded by the attending physicians for guidance of treatment choice. Image study including X-ray can only provide little additional information. The role playing by the routine X-ray examination is for ruling out the degenerative disease, or traumatic osteoarthritis caused by previous trauma. Although physical examination and history taking is noninvasive in nature, it is not scientifically precise due to various patients' characteristics of self-report. This drawback of subjectivity is especially annoying when patient has secondary gain of faking disease status, such as for asking vacation from company, or acquiring payment from insurance company. Therefore, how to diagnose the tennis elbow on solid scientific ground is thus an important issue.

To our knowledge, the tennis elbow has no gross pathological change like other traumatic injuries such as traumatic osteoarthritis, or tendon rupture. The pathological change is over microscopic level, so tennis elbow is often described as “microtear.” In electron microscopic level, it has been reported that plasma membrane tear is observed in fibrositis and tendinitis condition. However, it is almost impossible to obtain pathologic specimen during the treatment of tennis elbow. The pathology specimen can only be obtained from surgery, while operative treatment is very rare, because the operation is only for extremely refractory patients. In clinical practice, the conservative treatment modalities have been greatly improved in past years that almost every tennis elbow can be treated successfully. In our clinics, nonoperative treatments for tennis elbow include oral medication (NSAIDs, nonsteroidal anti-inflammatory drug), local steroid injection, rehabilitation, and ESWT (Extracorporeal Shock Wave Therapy). The majority of our patients receive local steroid injections, and the responsive rate is quite satisfactory. During treatment, tenderness experienced by patient is recorded as guideline for disease convalescence. Sometimes, the treatment plan must be changed based on the tenderness progression condition. However, tenderness is a subjective feels, it may be affected by patient's personality, social economic status, and culture backgrounds. If the surface EMG can be an objective measurement for tennis elbow disease, the treatment planning will have more information to count on [23–26].

Surface EMG records the electrical potential of muscle and nerves during action of motor units. However, the action potential of motor units is very complicated in time and spatial domain. Taking wrist extension as one example, this motion needs coordination of many muscles. Both agonist and antagonist muscle groups are coordinated. Besides, every single muscle in the same functional group also activated in different time. Therefore, the surface EMG signal we recorded is the spatial sum of multiple nerves and muscles. The waveform is complicated as shown in Figures 4 and 5. It is hard to differentiate between flexion and extension stages. The sEMG signals generated by motor units are nonstationary and multicomponent in nature and, consequently, the optimal signal analysis method remains to be elucidated. In this study, every sEMG signal is bounded with a VAS score representing the severity of tenderness by patient self-report. The severity of tenderness is evaluated using the visual analog score (VAS) as shown in Figure 1. Useful features from the sEMG signals are then correlated with the VAS scores.

To quantify the relationship between tenderness of tennis elbow and EMG voltage intensity measured from the epicondyle, which identify a more specific EMG relationship [23–26]. We have to develop the logistic and the probit DP of EMG signal measured from the epicondyle for tenderness of tennis elbow endpoint. The equipment for acquiring the sEMG signal is shown in Figure 3. This demonstrated the wrist in (a) full flexion and (b) full extension position. The electrodes used are the same as standard electrocardiogram leads. The electrode has one sticky surface and one side of metal buttock for conducting wire attachment. The sticky surface of electrodes will be degenerated after several times of use. Besides, when skin is wet or hairy, it is also difficult to attach the electrodes firmly to skin surface. At this situation, cleaning of the sticky surface with water is of some help. Otherwise, discard the old electrode will be a reasonable choice.

The electrodes are placed as shown in Figure 2. In fact, where to place the electrode is of vital importance, because this will affect the potential vector sum of the signal. As we know, different anatomical position of electrodes in EKG will generate different lead signals, such as aVF, Lead I, and V1-6. In this study, only one “lead” is recorded for sEMG signal, that is, ECRB muscle belly stands as positive, radial styloid as negative, and ulnar styloid as ground reference. This design is reasonable and gives consistent results.

In Figure 5(a), a signal from normal control is shown. The signal consisted of ten complete cycles, lasting about thirteen seconds. The detailed view of one cycle in Figure 5(a) is shown in Figure 5(b). From Figure 5, a central lump can be discerned from the wave form of sEMG signal. However, there is indentation over central lump region. The diseased wave form from a patient of VAS score 7.0 is shown in Figures 6(a) and 6(b). Comparing Figures 5 and 6, it can be observed that the lump is more flatten in diseased group. The baseline of diseased waveform is also more versatile than normal group. This result is compatible with previous report. In our preliminary studies, the earlier activation of muscle and more dissipative action potential is observed. However, how the extract useful feature from sEMG waveform remains a big challenge. The difficulty we encountered raise from two places: one is the noise when acquiring the signal. The other is the versatility of the waveform and electric amplitude. The noise during data acquisition is not from surrounding environment, but from the contact surface of skin and electrodes, and from the voltage amplifier circuits. The versatility of waveform comes from the intrinsic difference of skin resistance, muscle activation sequence, and motor unit mass. The variability of waveform also comes from the patient suffering from tennis elbow. During data acquisition, they are unwilling to do full range of motion because of pain. In tennis elbow patient, extension exercise is limited more than the flexion. Besides, both flexion and extension exercise are forbidden than the normal subjects. By the same reason, the statistical parameters and zero crossing methods all cannot obtain satisfactory results.

In order to solve the problem of signal versatility especially over the lump region, we have to adopt nonparametric methods such as AVTH and EUV. The mean AVTH curves for patients with/without tennis elbow are shown in Figure 7. It can be seen that the normal curve has lower intensity than diseased curve; thus, the curve is more concentrated over left side. The diseased curve is more flattened because it contains more high intensity “lumps” as shown in Figure 5. The benefit of AVTH is evident: the diseased group and normal control can be separated clearly. The power of AVTH comes from its normalization of intensity in the whole area of the EMG signal occupied. This normalization process is important because the intensity and the area varied from signals to signals. The second merit of AVTH is that the histogram equalizes the variation of the lump position. Some diseased patient has earlier lump position, while the other has later lump position. This feature of the diseased signal is very difficult to deal with conventional time domain method, while in AVTH this problem is not that annoying.

However, the AVTH curve as shown in Figure 7 needs to be further transformed for use as a feature. EDV took the information from the AVTH and then to be used as the EMG intensity feature to fit the logistic regression and the probit DP models. Further, we redefined the diseased group. VAS score larger than 3.0 is labeled “tennis elbow” and VAS score less than 3.0 as “normal subjects”; then, the dependent variable now becomes a nominal scale of two status. On this ground, the probit and logistic regression can be performed. The logistic regression model is built and shown in Figure 8(a). The probit model is calculated and shown in Figure 8(b). In fact, because the dependent variable has become binomial, these two models should be similar. The choice of probit versus logit model depends largely on individual preferences. Finally, the system performance is shown in Table 3. The overall performance for the DP models for the VAS 3+ tenderness of tennis elbow in terms of the AUC, Hosmer-Lemeshow test, Brier score, and* R* ^2^ was satisfactory with the expected values.

In this study, we found the TV_50_ ≥ 153 mV and TV_25_ ≥ 103 mV, those thresholds could prove especially advantageous in the treating of tennis elbow, in which regions require treatment and for which the tenderness affecting the patient's quality of life. The TV_50_ and TV_25_ values can provide guidance in setting treatment decisions and for predicting the recovery of the VAS 3+ tenderness of tennis elbow during treatment. The results of this study indicate that surface EMG provides useful data in evaluating severity of tennis elbow. There is a potential limitation of this study; namely, the number of patients evaluated was small, so a larger study sample is probably needed to demonstrate the independent association of these DP models with VAS 3+ tenderness of tennis elbow. The standard voltage for EMG acquisition unit needs to be set for further investigation. The innovative device used and the algorithm proposed can assist the clinician and other researchers to further be investigated in this issue.

## 5. Conclusion

The features of sEMG in lateral epicondylitis patients can be extracted with an innovative algorithm we proposed. The prediction model can be formed using logistic and probit techniques. When the EUV ≥ 153 mV, the DP of the patient is greater than 50%. The logistic and the probit models are valuable tools to predict the DP of VAS 3+ tenderness of tennis elbow. Therefore, sEMG is a valuable tool for diagnosis of tennis elbow.

## Figures and Tables

**Table 1:** Demographic data of subjects enrolled in this study.

Item	Status	Numbers (%)
Gender	Male	60 (76.9)
	Female	18 (23.1)

Age	≤39	54 (69.2)
	40–60	18 (23.1)
	>60	6 (7.7)

VAS score	0	51 (65.4)
	1-2	6 (7.7)
	3-4	2 (2.6)
	5-6	12 (15.3)
	7-8	5 (6.4)
	9-10	2 (2.6)

VAS 3+ tenderness	Yes	21 (26.9)
	No	57 (73.1)

VAS: Visual Analog Score.

**Table 2:** Diseased probability (DP) fitted parameters.

DP model	TV_50_ (95% CI) mV	*γ*_50_ or *m* (95% CI)	TV_25_ (95% CI) mV
Logistic	153.05 (136.31–169.79)	0.84 (0.78–0.90)	103.10 (86.36–119.84)
Probit	155.67 (138.93–172.41)	0.54 (0.49–0.59)	98.97 (67.57–101.05)

DP: diseased probability; CI: confidence interval; TV_50_: the threshold voltage predicting a 50% risk of disease; TV_25_: the threshold voltage predicting a 25% risk of disease; *m*: a unitless probit model parameter for describing the slope of the DP curve; *γ*_50_: a logitic model parameter for normalized slope of the DP curve.

**Table 3:** System performance evaluation.

	Logistic	Probit
AUC	0.84 (0.74–0.94)	0.84 (0.74–0.94)
Accuracy	0.78	0.78
Brier (scaled)	0.34	0.39
*R* ^2^ Nagelkerke	0.36	0.28
HL	0.29	Na
*χ* ^2^ (*P*)	0.27 (0.60)	0.32 (0.57)
NPV-TV_50_	0.83	0.84
NPV-TV_25_	0.90	0.93
AIC	66.70	68.64

AUC: area under the receiver operating characteristic curve; HL: Hosmer-Lemeshow test; NPV: negative predictive value; *χ* ^2^(*P*): a chi-square goodness-of-fit test; *P* value of >0.05 indicates lack of statistical difference between predictive risk and the observed outcome.
